# Multivariate Meta-Analysis of Brain-Mass Correlations in Eutherian Mammals

**DOI:** 10.3389/fnana.2016.00091

**Published:** 2016-09-30

**Authors:** Charlene Steinhausen, Lyuba Zehl, Michaela Haas-Rioth, Kerstin Morcinek, Wolfgang Walkowiak, Stefan Huggenberger

**Affiliations:** ^1^Department II of Anatomy, University of CologneCologne, Germany; ^2^Biocenter, University of CologneCologne, Germany; ^3^Jülich Research Centre, Institute of Neuroscience and Medicine (INM-6) and Institute for Advanced Simulation (IAS-6) and JARA BRAIN Institute IJülich, Germany; ^4^Department of Anatomy III (Dr. Senckenbergische Anatomie), Goethe University of Frankfurt am MainFrankfurt am Main, Germany

**Keywords:** eutheria, mammals, brain mass, brain size, EQ, cluster analysis, trophic level

## Abstract

The general assumption that brain size differences are an adequate proxy for subtler differences in brain organization turned neurobiologists toward the question why some groups of mammals such as primates, elephants, and whales have such remarkably large brains. In this meta-analysis, an extensive sample of eutherian mammals (115 species distributed in 14 orders) provided data about several different biological traits and measures of brain size such as absolute brain mass (AB), relative brain mass (RB; quotient from AB and body mass), and encephalization quotient (EQ). These data were analyzed by established multivariate statistics without taking specific phylogenetic information into account. Species with high AB tend to (1) feed on protein-rich nutrition, (2) have a long lifespan, (3) delayed sexual maturity, and (4) long and rare pregnancies with small litter sizes. Animals with high RB usually have (1) a short life span, (2) reach sexual maturity early, and (3) have short and frequent gestations. Moreover, males of species with high RB also have few potential sexual partners. In contrast, animals with high EQs have (1) a high number of potential sexual partners, (2) delayed sexual maturity, and (3) rare gestations with small litter sizes. Based on these correlations, we conclude that Eutheria with either high AB or high EQ occupy positions at the top of the network of food chains (high trophic levels). Eutheria of low trophic levels can develop a high RB only if they have small body masses.

## Introduction

One of the core questions of neurobiology is how some groups of animals such as primates, elephants, and whales have evolved remarkably large brains (Haug, 1987; Marino, 1998; Roth and Dicke, 2005). Size differences of whole brains were interpreted as an adequate proxy for subtler differences in anatomy and function (Jerison, 1973; Stephan et al., 1988; van Dongen, 1998; Lefebvre et al., 2004, 2007, 2013; Marino et al., 2007). Because larger animals have larger brains (Harvey et al., 1980) but the functional or cognitive capacities of their brains are not necessarily greater, the use of absolute brain mass (AB) to compare different species of varying body size is limited. The correlation between AB and body mass (BM) does not increase in a linear fashion, which means that animals of smaller sizes have a proportionally higher relative brain mass (RB) than larger animals (van Dongen, 1998). For these allometric reasons, the encephalization quotient (EQ), a parameter indirectly dependent on the size of a body, is an useful metric for comparing brain sizes among mammals of different size (Baron, 2007). The EQ is defined as the ratio of the actual mass of the brain to the expected brain mass given by the body mass (van Dongen, 1998).

Several comprehensive studies show that a plain correlation of brain size and its functional capacity is not valid since subtler morphological and physiological differences may explain individual adaptations of cognitive capacities (Hof et al., 2000; Manger, 2005; Roth and Dicke, 2005; Douglas and Martin, 2007; Elston, 2007; Butti et al., 2009; Shultz S. and Dunbar R. I. M., 2010; Kern et al., 2011; Dicke and Roth, 2016). Brain size may not be the main factor of functional capacities (Harrison et al., 2002; Herculano-Houzel et al., 2007; Krubitzer, 2007; Sarko et al., 2009). The increase of brain volume is usually paralleled by a structural differentiation that may result in variation in the relative size of distinctive parts of the brain (Starck, 1979; Baron et al., 1996; Voogd et al., 1998; Allman, 1999; Glickstein et al., 2007; Reep et al., 2007; Sarko et al., 2009; Shultz S. and Dunbar R. I. M., 2010). These and other authors (Elston et al., 2001; DeFelipe et al., 2002; Elston, 2002, 2007; Nedergaard et al., 2003; Sarko et al., 2009; Raghanti et al., 2015) show that the complexity and diversity of (micro-) circuits and principal neurons as well as the number of glial cells may be a main factor to influence the “computational power” of mammalian isocortices. The specification, the arrangement, and the numbers of neurons, glia cells, and neuronal connections usually vary substantially in mammalian brains (Krubitzer, 1995, 2007; Hof et al., 2000; Kaas, 2000; Elston et al., 2001; DeFelipe et al., 2002; Elston, 2002; Harrison et al., 2002; Krubitzer and Kaas, 2005; Manger, 2005; Roth and Dicke, 2005; Herculano-Houzel et al., 2007; Sarko et al., 2009; Herculano-Houzel, 2011; Homman-Ludiye and Bourne, 2014; Dicke and Roth, 2016). Comparisons of detailed neuroanatomy would thus contribute considerably to our understanding of the evolution of the mammalian brain (DeFelipe et al., 2002; Elston, 2007; Herculano-Houzel et al., 2007; Dechmann and Safi, 2009; Sarko et al., 2009; Kern et al., 2011).

Apart from the above mentioned factors, which have an immediate effect on the information processing capacities, the ecological niche of a given species is often reflected in the morphological specification of the brain and slight differences in its structure reflect a variety of eco-ethological adaptations (Stephan et al., 1988; Voogd et al., 1998; Oelschläger, 2008). Nevertheless, a number of studies described critical biological factors influencing relative mass of the brain and the EQ, respectively (Barton and Dunbar, 1997; Dunbar, 1998, 2003, 2009; Changizi, 2003; Lefebvre et al., 2004, 2013; Manger, 2006; Shoshani et al., 2006; Shultz and Dunbar, 2006; Dunbar and Shultz, 2007; Hart et al., 2008; Finarelli, 2009; Shultz S. and Dunbar R., 2010; Navarrete et al., 2011; Boddy et al., 2012; Fitzpatrick et al., 2012; McNally et al., 2012; Arsznov and Sakai, 2013). The large body of older references regarding this topic is comprehensively discussed by van Dongen (1998). These studies focused on the volumes of whole brains (Healy and Rowe, 2007) assuming that convergent evolution shaped the whole brain of several mammalian taxa through selection arising from similar ecological requirements and natural history (de Winter and Oxnard, 2001; Kaas, 2002; Lefebvre et al., 2007; Montgomery et al., 2013; Cozzi et al., 2014). Among these factors are:

1. Physiological parameters such as sexual maturity, life expectancy, adipose depots, and metabolism rate (including thermogenesis),
2. Developmental parameters such as gestation period, length of neurogenetic period, nursing period, pregnancy, menopause, number, and level of physical development of the offspring,
3. Ecological parameters such as nutrition and circadian rhythm, and
4. Behavioral parameters such as propagation patterns, social behavior, sexual selection, and higher cognitive abilities such as learning, play behaviors, and innovation (see references above).

Comparison of the studies mentioned above revealed that each considered only a few parameters analyzed in a univariate fashion. Furthermore, these studies were restricted to few genera and species and only a minor part of these analyzed the connection of brain size and cognitive abilities. Remarkably, the recent literature has largely considered only very special parameters of cognitive abilities, e.g., tool use in primates and birds (Reader and Laland, 2002; Iwaniuk et al., 2005; Emery, 2006; Deaner et al., 2007; Lefebvre et al., 2013). Apart from that the focus has concentrated mainly on the analysis of the volume of the cerebral cortex (Barton and Dunbar, 1997; Dunbar, 1998, 2003, 2009; Voogd et al., 1998; Dunbar and Shultz, 2007; Shultz S. and Dunbar R., 2010). Other cerebral characteristics, such as the number of neurons, the intensity of cortical folding, and especially the relative sizes of distinctive parts of the brain, would have been interesting for comparative studies of brain size. However, such cerebral dimensions have currently only been analyzed for a very limited number of genera and species (Healy and Rowe, 2007; Reep et al., 2007; Herculano-Houzel, 2011).

To overcome some of the restrictions of the former studies mentioned above we compared data of a large number (115) of mammalian species (Eutheria, Placentalia) representing 14 orders; a total of up to 21 orders of Eutheria were defined so far (Storch and Asher, 2015). In contrast to most recent studies cited above, we analyzed the mass of the whole brain instead of brain parts and functional systems because these data were available in literature for a large number of species. Moreover, the measurement of the whole brain is less biased by a specialization of a single system such as hypertrophy of a single sensory system (Willemet, 2012). This paper focuses on Eutheria because only few data are available concerning the brain mass of Protheria and Metatheria species. We made use of multivariate analyses based on far-reaching literature records. The resulting meta-analysis showed which biological parameters, independent of any systematic considerations, may be correlated with a large AB, a large RB, as well as a large EQ.

## Materials and methods

### Species and parameters

**Figure 1:**
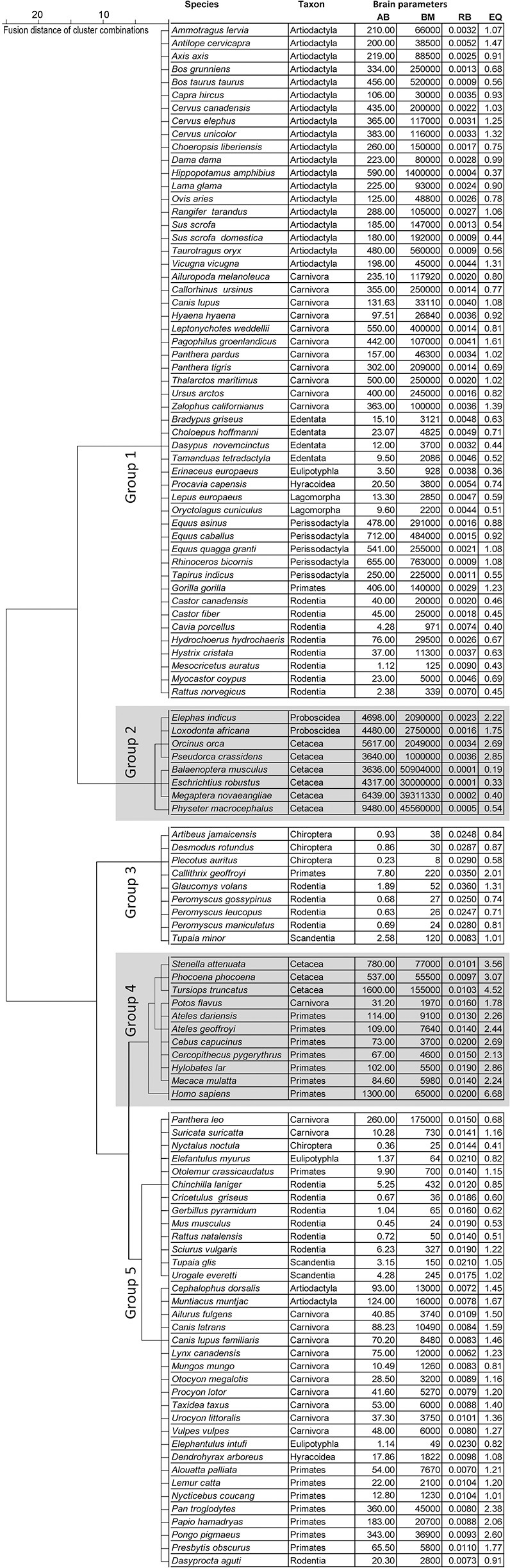
**Dendrogram of the cluster analysis for the three brain dimensions absolute brain mass (AB), relative brain mass (RB), and encephalization quotient (EQ) subdivided into five endclusters (groups 1–5)**.

We collected data from the literature (Stewart, 1902; Pettit, 1905; Crile and Quiring, 1940; Kozima, 1951; Jansen, 1953; Oboussier and Schliemann, 1966; Ridgway et al., 1966; Sigmund, 1968; Gihr and Pilleri, 1969; Pilleri and Busnel, 1969; Gruenberger, 1970; Pirlot and Stephan, 1970; Oboussier and Möller, 1971; Oboussier, 1972; Pilleri and Gihr, 1972; Ebinger, 1974; Sacher and Staffeldt, 1974; Harper and Maser, 1976; Meester and Setzer, 1977; Radinsky, 1978, 1981; Kamiya and Pirlot, 1980; Osborne and Sundsten, 1981; Ridgway, 1981; Hofman, 1982; Nowak and Paradiso, 1983; Schwerdtfeger et al., 1984; Gittleman, 1986; Stephan et al., 1988; Eisenberg, 1989; Puschmann, 1989; Tarpley and Ridgway, 1994; Gingerich, 1998; Dahlheim and Ridgway, 1999; Marino et al., 2000; Perrin et al., 2002; Wilson and Reeder, 2005; Wund and Myers, 2011) concerning the brain and body masses of 1180 adult Eutheria grouped in 115 species of 14 orders (Storch and Asher, 2015) (the monophyletic taxon Cetartiodactyla was divided into its former groups Artiodactyla and Cetacea due to their different annidations; Frey et al., 2015; Huggenberger and Klima, 2015): (1.) Artiodactyla (21 species), (2.) Carnivora (24 species), (3.) Cetacea (9 species), (4.) Chiroptera (4 species), (5.) Hyracoidea (2 species), (6.) Lagomorpha (2 species), (7.) Lipotyphla (1 species), (8.) Macroscelidae (2 species), (9.) Perissodactyla (5 species), (10.) Primates (17 species), (11.) Proboscidea (2 species), (12.) Rodentia (19 species), (13.) Scandentia (3 species), (14.) Xenarthra (4 species). The selection of species depended on the availability of brain masses and biological data (Figure 1; see Table S1). For this reason not every order is represented with an equal number of species. Data of AB and BM were taken only when both values were from the same individual. The statistical means of AB and BM were calculated when data from more than one individual were available in literature. The original data taken from the literature were not further corrected for potential inaccuracies such as fixation artifacts or differences in the usage of equations for body mass calculations of large mammals.

The relative brain mass (RB) was calculated as the quotient from absolute brain mass (AB) and body mass (BM) (van Dongen, 1998).

The data for AB and BM show a logarithmical relationship, which can be depicted in the following equation:

$$\begin{array}{l} \text{log }\left(\text{AB}\right)=\alpha *\text{log }\left(\text{BM}\right)+\text{log }\left(\text{k}\right) \end{array}$$

where α is the slope of the regression line and k its intercept.

With this power function the encephalization quotient (**EQ**) of every species was calculated:

$$\begin{array}{l} \text{EQ}={\text{E}}_{\text{t}}/{\text{E}}_{\text{e}}, \end{array}$$

where E_t_ is the actual brain mass and E_e_ is the expected brain mass according to the power function (Voogd et al., 1998).

Additionally, the following biological parameters were taken from literature (see above): (1.) sexual maturity in days; (2.) maximum lifespan in years; (3.) gestation period in days; (4.) nursing period in days; (5.) litter size; (6.) frequency of pregnancies (pregnancies per year); (7.) period of postnatal eye opening in days (eye opening); (8.) group size; (9.) mean number of female sexual partners during a 10 years period (female partners); and (10.) mean number of male sexual partners during a 10 years period (male partners); (11.) percentage of raw protein in dry mass of nutrition (protein; Table 1); (12.) percentage of raw fibers in dry mass of nutrition (fiber; Table 1); and (13.) activity period in hours during daytime (circadian activity).

**Table 1:** **Definition of nutrient values for food types (see Table S1) of Eutheria mentioned in this study (Puschmann, 1989; Subcommittee on Laboratory Animal Nutrition Committee on Animal Nutrition Board on Agriculture National Research Council, 1995; Committee on Animal Nutrition Ad Hoc Committee on Nonhuman Primate Nutrition National Research Council, 2003; Dillizer, 2009)**.

**Food type**	**(Raw) protein**	**Raw fat**	**(Raw) fiber**	**Carbon-hydrates**	**Raw ash**
1 – Mere carnivore	60	20	1	1	18
2 – Piscivore	61.3	2.6	0.3	1	22
3 – Misc. carnivore	40	5	3.5	50	1.5
4 – Omnivore	20	9.5	32	8.6	10
5 – Insectivore	48.7	7.5	5.3	1	17.1
6 – Folivore	13	3.5	55	10	10.5
7 – Graminivore	11	1.9	29.3	28.3	6.6
8 – Foli- & graminivore	12	2.7	42.2	19.2	8.6
9 – Frugivore	7.5	5	40	15	2.5
10 – Herbivore	14.7	3.5	18	4.9	5.6

*Nutrient values are defined in percent of dry matter. Two carnivorous types were set apart from each other: mere carnivores relating to the family of Felidae and miscellaneous carnivores are Canidae, as well as Thalarctos maritimus and Taxidea taxus among others (see Table S1)*.

In order to compare all 13 biological parameters with the same multivariate statistical procedure, all variables were converted to a metric system. For this purpose, masses were specified in grams, temporal data in days and the frequency of pregnancies in births per year as well as in offspring per litter. To analyze the factor of maximum lifespan, data from the wild and from zoos were combined and the mean value was used. If only one value was available, either the wild or zoo keeping was taken into account. Average values were calculated from the sexual maturity ages of males and females. Group size (in the case of herds: the group size of recorded close social contact) was included as a numeric value to represent a proxy of social structures (Nowak and Paradiso, 1983; Wund and Myers, 2011).

For a numerical consideration, ten different food types were defined by the percentage of dry matter of singular nutrients (Table 1). Nutrient percentages were derived from available literature (Puschmann, 1989; Subcommittee on Laboratory Animal Nutrition Committee on Animal Nutrition Board on Agriculture National Research Council, 1995; Committee on Animal Nutrition Ad Hoc Committee on Nonhuman Primate Nutrition National Research Council, 2003; Dillizer, 2009). Values for facultative carnivores (e.g., Canidae, which consume a minimal percentage of vegetable food) and herbivores were oriented toward the specifications given for zoo animals and by the animal-feed industry (http://futter.wildvogelpflege.de; http://www.grau-gmbh.de; http://www.jr-farm.de/; http://www.hundeland.de/) (Table 1). Accordingly, these types of nutrition were used for quantitative comparisons and do not represent measured values of food intake of the respective species in the wild. However, this metric classification enabled us to draw general conclusions concerning food intake of the respective eutherian species.

The types of circadian activity were allocated to numeric values which represent the estimated number of hours per day that an animal is potentially active in broad daylight of a standardized 12 h daytime day (Nowak and Paradiso, 1983; Wund and Myers, 2011). For diurnal Eutheria, the circadian activity was determined by 12 h, crepuscular animals are ~3 h active, crepuscular to nocturnal animals only 1 h, and nocturnal Eutheria are not active during daytime (0 h). In some species, the “activity differed” because they may adjust their day or night time activity to the corresponding habitat or food resources. Here, 6 h of circadian activity was arbitrarily assigned.

To depict the mating system of the Eutheria numerically, the number of possible different male and female sexual partners per 10 pregnancies was calculated. In this way we could differentiate between monogamy and polygamy of seasonal mating systems: A monogamous male animal would only have one sexual partner during these ten mating times, a seasonally mating animal, 10 possible partners, a polygynously reproducing mammal, a number of partners equaling the size of a harem. A polygynandric animal would have as many female sexual partners as are available within the group. Concerning male sexual partners, the number within the harem goes down to one, while the number of partners in the other mating systems equals the number of female mating partners. The individual specifications of mating partners are described in Table S1.

### Cluster analysis of brain parameters

For the cluster analysis the brain parameters AB, RB, and EQ were normalized (quotients from the respective single values and the corresponding peak value). To check for potential correlations between these brain parameters the correlation factor after Pearson was calculated using Excel 2013 (Microsoft Corporation, Redmond, Washington, DC, U.S.A.). Then the clusters were created based on the conservative Ward-method using SPSS 19.0 (IBM Corporation, Armonk, NY, U.S.A.), since it is regarded a reliable fusion algorithm and because it forms groups of relatively equal size (Backhaus et al., 2008). As a means of specifying the distance dimension the squared Euclidean distance was chosen.

We selected final clusters (groups) after compiling a dendrogram. The selection of the number of these groups was based on the facts that: (1) the fusion distances for the compiling of the clusters were relatively high concerning a higher number of groups; and (2) groups were not comprised of only one species. Those could not have been analyzed in the following discriminant analysis. For a graphic differentiation of the groups their mean values of the standardized group parameters AB, RB, and EQ were depicted with their corresponding standard deviation. To validate for robustness of the cluster analysis a discriminant analysis of the final clusters were performed using the Wilks' Lambda-method SPSS 19.0.

### Discriminant analysis of the biological parameters

The discriminant analysis is the central statistic procedure of this study. Using the Wilks' Lambda-method of SPSS 19.0, we analyzed how the cluster groups (see above) were separated by the various normalized biological parameters (quotients from the respective single values and the corresponding peak value). Those biological parameters which contained a discriminatory power within the discriminant function were depicted as mean values of the normalized data set.

## Results

### Relative brain mass and encephalization quotient

**Figure 2:**
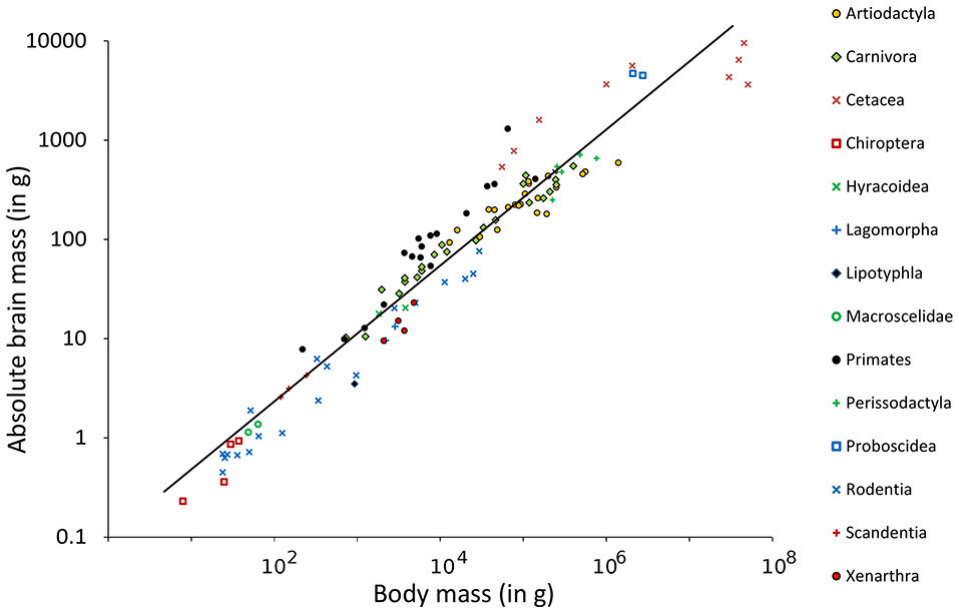
**Double logarithmic plot of absolute brain mass (AB) above body mass (BM) including regression line of 115 species of Eutheria arranged by order relationships (Figure 1)**.

The Southern flying squirrel *Glaucomys volans* had the highest relative brain mass (RB) found in any of the mammals in this sample with a brain making up of 3.6% of the body mass (Figure 1). The blue whale *Balaenoptera musculus*, in contrast, had only 0.007% and thus the smallest brain in relation to its body size. *Homo sapiens* had a similar RB (about 2%) as some rodents and would, for example, be comparable in this regard to the house mouse *Mus musculus* whose brain makes up 1.9% of the body mass. Related groups whose body masses differed from each other also differed in their RB, a result which conforms to the expected brain and body mass allometry and its function of the regression line calculated in a double logarithmic diagram (Figure 2). The resulting power function was

$$\begin{array}{l} \text{AB}=0.0951*\text{B}{\text{M}}^{0.688}. \end{array}$$

Using this function, the EQ of every single animal was calculated (see Table S1). For the species mentioned in the examples above, the mean EQ of the Southern flying squirrel was 1.3. The EQ of the blue whale was 0.19 and thus still the lowest EQ in this study. The EQ of man was 6.68 and thus 12.6 times higher than the EQ of the house mouse (0.53) and at the same time the highest EQ of all animals in this study.

The dependency of the three brain parameters AB, RB, and EQ were low which was shown by the correlation factors. For the normalized parameters these factors were −0.28 for AB vs. RB, 0.11 for AB vs. EQ, and 0.22 for RB vs. EQ.

### Cluster analysis

A cluster analysis was used to group all species in this meta-analysis according to their brain parameters AB, RB, and EQ. Five groups could be separated: two relatively large groups with 52 (group 1) and 35 (group 5) species, respectively; and three smaller groups with 8 (group 2), 9 (group 3), and 11 (group 4) species (Figure 1).

**Group 1** contained nine orders of Euteria. Highest in number were the Artiodactyla with 19 of the 21 species (exceptions: the relatively small Indian muntjac *Muntiacus muntjak* and the bay duiker *Cephalophus dorsalis*). Moreover, there were four Xenarthra species, two Lagomorpha as well as 11 of 23 Carnivora species. The latter included Pinnipedia, Ursidae (including the herbivorous giant panda *Ailuropoda melanoleuca)*, the leopard *Panthera pardus* and the tiger *Panthera tigris* from the family of Felidae, the wolf *Canis lupus* and the striped hyena *Hyaena hyaena*. In addition, group 1 contained 19 representatives from the group of Rodentia, including guinea pigs (Caviidae), the coypu *Myocastor coypus*, two species from the beaver family (Castoridae), the golden hamster *Mesocricetus auratus*, the brown rat *Rattus norvegicus*, and the crested porcupine *Hystrix cristata*. Group 1 also contained the European hedgehog *Erinaceus europaeus* from the family Insectivora (Lipotyphla), the rock hyrax *Procavia capensis* (Hyracoidea), and the gorilla *Gorilla gorilla*, which was the only primate in this group. Note that the larger species of the respective taxa were contained in group 1; e.g., the gorilla *G. gorilla* and the European beaver *Castor fiber*. The golden hamster (BM 0.125 kg, AB 1.12 g) and the brown rat (BM 0.34 kg, AB 2.38 g) were exceptions in this respect because the average BM in this group was 160 ± 34 kg, the average AB was 238 ± 28 g.

**Group 2** comprised large mammals (BM 21708 ± 8280 kg, AB 5288 ± 734 g), the two Proboscidea and six larger representatives of the Cetacea.

In contrast to group 2, **group 3** comprised representatives of four orders of relatively small mammals (BM 0.06 ± 0.02 kg, AB 1.8 ± 0.8 g). It contained three Chiroptera species, the pygmy treeshrew *Tupaia minor* (Scandentia), the white-headed marmoset *Callithrix geoffroyi* from the primates as well as the Southern flying squirrel *G. volans* and the three *Peromyscus* species from the rodents.

**Group 4** contained three representatives of new world monkeys (Platyrrhini), *Cebus capucinus* and the spider monkeys *Ateles dariensis* and *A. geoffroyi*, three smaller (rhesus macaque *Macaca mulatta*, vervet monkey *Cercopithecus pygerythrus*, the lar gibbon *Hylobates lar*), and one larger representative of the old world monkeys (Catarrhini; man *H. sapiens*). In addition, three smaller representatives of the Cetacea, the spotted dolphin *Stenella attenuata*, the bottlenose dolphin *Tursiops truncatus* and the harbor porpoise *Phocoena phocoena*, as well as one representative of carnivores, the kinkajou *Potos flavus*. The average BM of group 4 was 36 ± 17 kg, the average AB was 436 ± 190 g.

**Group 5** contained 8 orders and many representative orders from group 1 such as the Artiodactyla with the Indian muntjac *M. muntjak* and the bay duiker *C. dorsalis*. The remaining carnivore representatives were the Canidae, the raccoon *Procyon lotor*, the Herpestidae (*Suricata suricatta, Mungos mungo*), the American badger *Taxidea taxus* as well as the Canada lynx *Lynx canadensis* and the lion *Panthera leo* from the family of Felidae. The Chinese hamster *Cricetulus griseus*, the red-rumped agouti *Dasyprocta aguti* and the long-tailed chinchilla *Chinchilla lanigera* represented the family of rodents in group 5. Primates belonging to group 5 were four Platyrrhini and four Catarrhini, including the Hamadryas baboon *Papio hamadryas*, the Bornean orang-utan *Pongo pygmaeus*, and the chimpanzee *Pan troglodytes*. The two Elephantulus species were the only representatives of the Macroscelidae and the insect-eating *Nyctalus noctula* represented the only species of the Chiroptera in this group. The average BM of group 5 was 11 ± 5 kg, the AB 60 ± 16 g.

**Figure 3:**
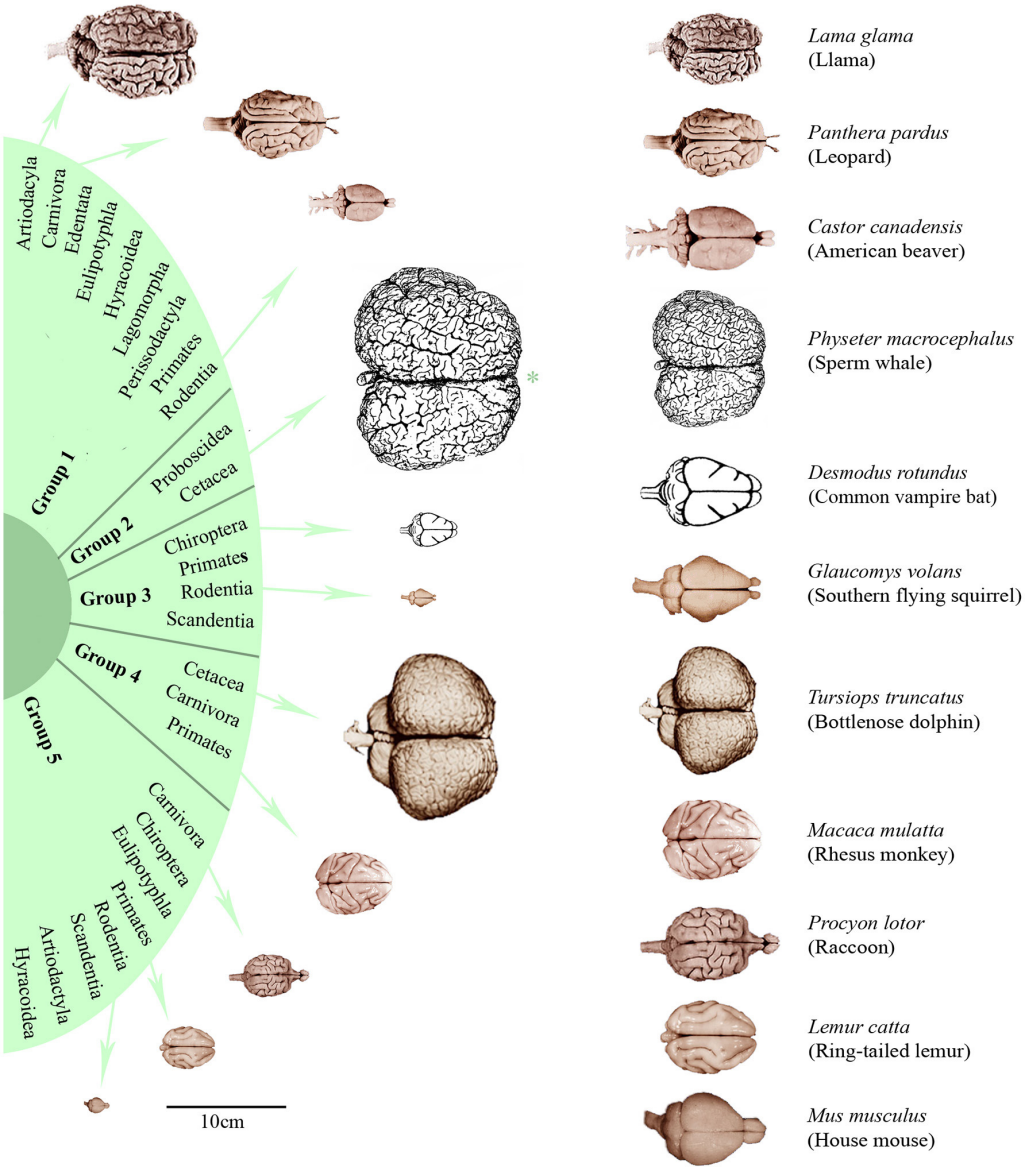
**Dorsal brain views of representative eutherian species arranged according to the dendrogram shown in Figure 1**. On the left, the brains were aligned in scale (^*^except the sperm whale brain which is only half of its scale) and, on the right, scaled to the same total length. Brain photographs were taken from the University of Wisconsin and Michigan State Comparative Mammalian Brain Collections (www.brainmuseum.org), the sperm whale brain was depicted after Kozima (1951) and the common vampire bat brain after Baron et al. (1996).

**Figure 4:**
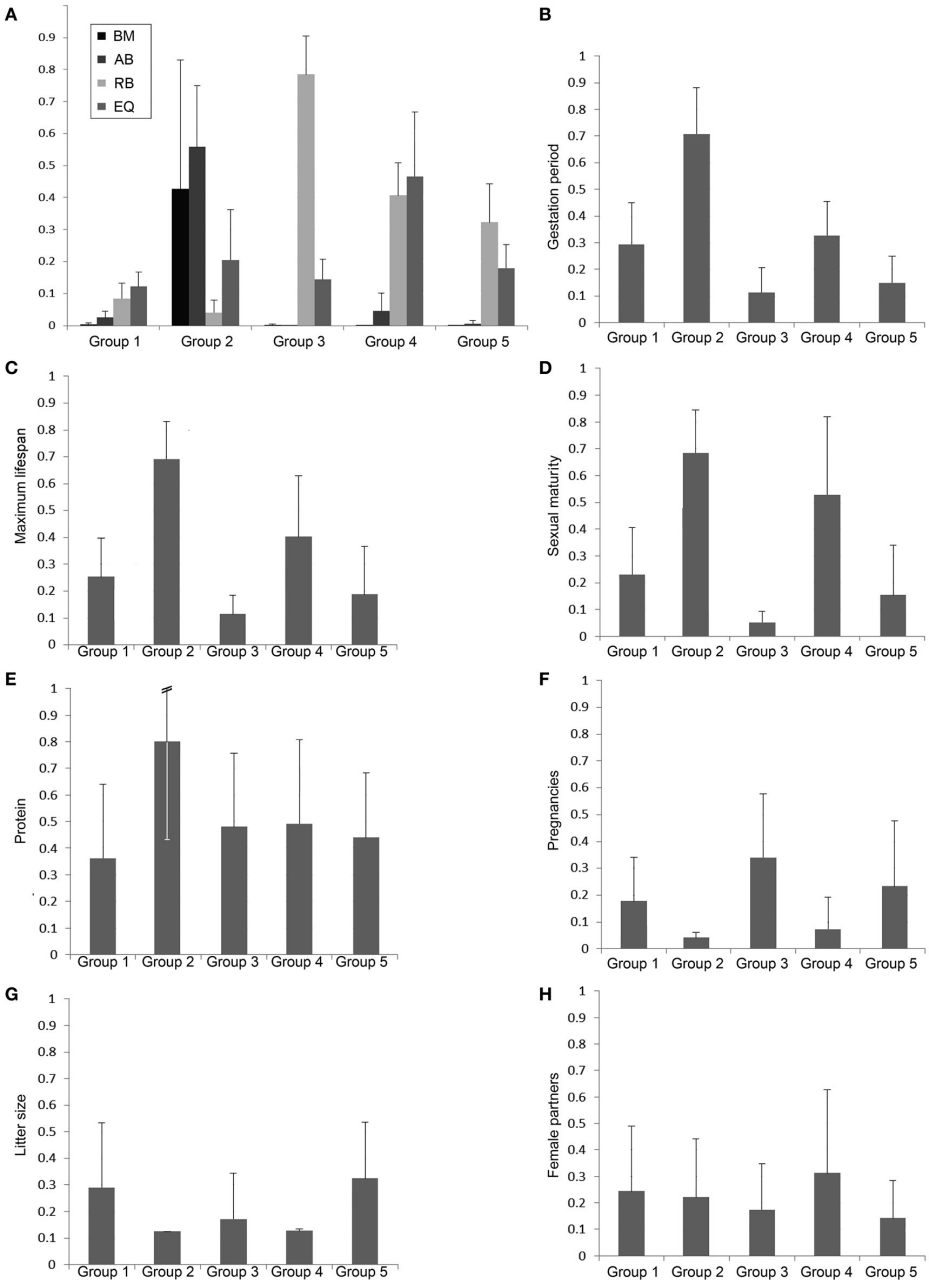
**Bar charts of normalized group mean values and standard deviations of the body mass (BM), absolute brain mass (AB), the relative brain mass (RB), and the EQ (A) as well as the biological parameters with potential discriminatory impact of the groups 1–5 defined by the cluster analysis (B–H)**.

In order to characterize the groups with regard to their brain dimensions considered above, representative brains of each group are shown in scale and normalized to the same size in Figure 3. Moreover, their normalized mean values and standard deviations were compared. Figure 4A shows that group 1 was characterized by relatively low values in all three brain parameters. Group 2 had the highest AB (0.56), the lowest RB (0.04), and an average EQ value. Group 3 had the lowest AB (< 0.01), the highest RB (0.78), and a smaller EQ than group 2. Group 4 showed a mean AB, a high RB and the largest EQ mean value (0.47). Group 5 had a low AB, a mean RB and an EQ between groups 2 and 3. In conclusion, it is striking that, on average, groups 2, 3, and 4 each had a maximum brain parameter that set them apart from the other groups.

Accordingly, the discriminant analysis of the same three brain parameters separated these five clusters highly significantly (*p* > 0.001; see Tables S2–S4) and the robustness of these results were verified by the fact that 92.2% of the cluster members were correctly reclassified in the discriminant analysis (see Table S5).

### Discriminant analysis of the biological parameters

For the discriminant analysis, the normalized biological parameters were allotted to the five groups separated by the cluster analysis. Table 2 shows how distinctly the 13 biological parameters were isolated (univariately) between the specific groups. The U-statistics (Wilks' Lambdal; Backhaus et al., 2008) revealed a significant separation (*P* < 0.05) for the variables gestation period, sexual maturity, maximum lifespan, raw protein in dry matter of nutrition (protein), frequency of pregnancies per year, litter size, and number of female sexual partners (female partners; Table 2). However, the variable gestation period proved to be the best means of separation because it had the lowest Wilks' Lambda.

**Table 2:** **Results of the U-statistics (Wilks' Lambda-method)**.

**Biological parameter**	**Wilks' Lambda**	**Significance (P)**
Gestation period	0.474	<0.001
Sexual maturity	0.562	<0.001
Maximum lifespan	0.588	<0.001
Protein	0.864	0.003
Pregnancies per year	0.869	0.004
Litter size	0.903	0.023
Female partners	0.913	0.040
Nursing period	0.920	0.056
Male partners	0.927	0.077
Circadian activity	0.931	0.093
Fiber	0.937	0.122
Eye opening	0.974	0.576
Group size	0.984	0.777

*The biological parameters were ordered according to their significance values*.

Table 3 describes the criteria to evaluate the discriminant function. The column “% of variance” shows that the importance of the fourth discriminant function with a variance share of 3.2% was essentially smaller than the third discriminant function with 10%, the second with 19.6%, and the first with 67.2%. In this multivariate analysis, the first three functions significantly supported the separation of the groups (*P* < 0.05). For the re-classification, 67% of the originally grouped cases could be assigned to the groups they belonged to.

**Table 3:** **Criteria to evaluate discrimant functions of biological parameters**.

**Function**	**Eigenvalue**	**% of Variance**	**Significance**
1	1.900	67.2	0.000
2	0.553	19.6	0.000
3	0.283	10.0	0.035
4	0.092	3.2	0.513

*The discriminant functions 1–3 contribute significantly to the separation of the groups (p < 0.05). The column “% of Variance” shows that the impact of the discriminant function 4 is essentially less than the other functions*.

**Figure 5:**
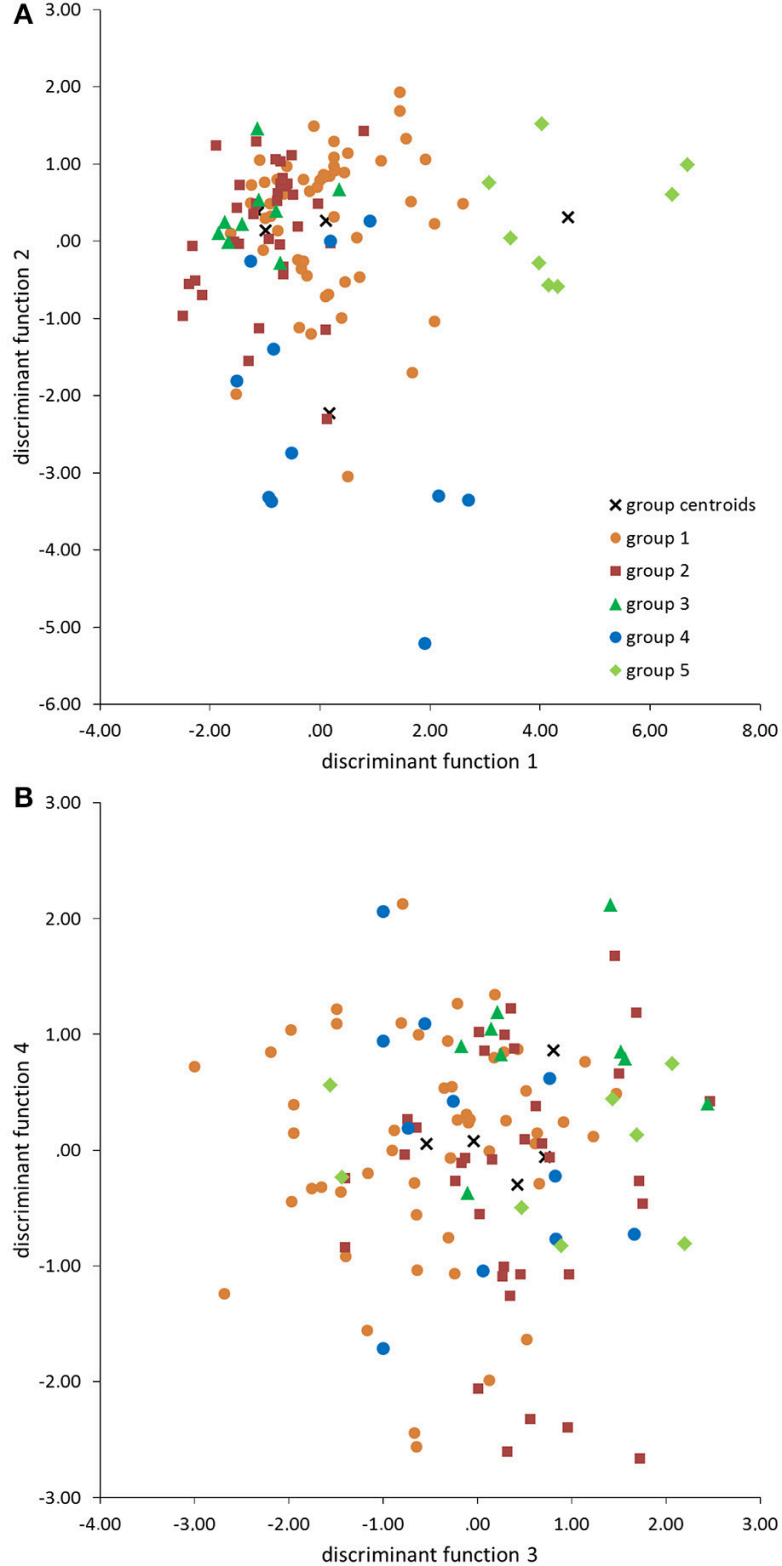
**Scatter plots of groups 1–5 defined by the cluster analysis (Figure 1) in the discriminant area of the biological parameters for the functions 1 and 2 (A), and 3 and 4 (B)**.

The standardized canonic discriminant coefficient in Table 4 shows the significance of the biological parameters within the first three discriminant functions (highest absolute value). Thus, the gestation period for functions 1 and 2, the maximum lifespan for function 1, the sexual maturity for function 2, and the percentage of raw protein in the dry matter of nutrition (protein) for function 3 had the largest discriminatory powers. From the plot of the discriminant functions, group 2 obviously separated itself from the other groups in the first discriminant function (Figure 5A). With regard to discriminant function 2, group 4 clustered apart from the other groups (Figure 5A). Groups 1 and 3 were separable by discriminant function 3, even if there was a relatively large overlap of data from these groups (Figure 5B). In the area of overlap the tendency of group 5 was to cluster (Figure 5B). As expected from the variance values (Table 3), the discriminant function 4 did not allow any further differentiation (Figure 5B).

**Table 4:** **Standardized canonic discriminant function coefficient for the significant discriminant functions 1–3 (Table 3) of the biological parameters**.

**Biological parameter**	**Function 1**	**Function 2**	**Function 3**
Gestation period	0.994^*^	0.732^*^	−0.437
Maximum lifespan	0.577^*^	0.439	0.755^*^
Litter size	0.378	0.328	−0.482
Protein	0.259	−0.049	0.984^*^
Pregnancies per year	0.098	0.233	0.521
Female partners	0.064	0.291	−0.727
Eye opening	0.053	0.418	−0.047
Sexual maturity	0.004	−1.371^*^	−0.375
Group size	−0.03	−0.033	0.292
Fiber	−0.141	−0.32	0.35
Male partners	−0.161	−0.692	0.22
Circadian activity	−0.185	−0.328	−0.221
Nursing period	−0.442	0.418	0.211

*The two maximum absolute values of each function were marked with an asterisk*.

In order to analyze the biological parameters that significantly separated the single groups via the discriminant analysis (U-statistics and discriminant coefficient), the normalized group mean values and their standard deviations were plotted. The Eutheria of group 2, which showed a maximum AB value and a minimal RB (Proboscidea and large Cetacea), had the largest gestation period (Figure 4B), the longest maximum lifespan (Figure 4C), the latest sexual maturity (Figure 4D), mainly fed on protein-rich nutrition (Figure 4E), and had the largest pregnancy intervals (lowest numbers of pregnancies per year) as well as the smallest litter size (Figures 4F,G).

The litter sizes in group 3 were about as small as in group 2 (Figure 4G), yet the eutherians from group 3 showed the highest numbers of pregnancies per year with a short gestation time (Figures 4B,F). These eutherians also had shorter lifespans and correspondingly reached sexually mature early (Figures 4C,D). The males from group 4 had the highest ratio of mating partners (Figure 4H). However, group 4 comprised species with similarly late sexual maturity (Figure 4D), with large pregnancy intervals, and small litter sizes (Figures 4F,G) as in group 2.

The mammals in groups 1 and 5 generally had no peak values with regard to these biological parameters. It is, however, noteworthy that species in group 1 consumed the least raw protein in the dry matter of the food (Figure 4E).

## Discussion

### Data composition

In our data set some values are referred only to a single individual, which was the case for, e.g., the blue whale *B. musculus* (Jansen, 1953). However, it was shown for some mammalian groups that there are high intraspecific and sex specific variations regarding brain mass and body mass (Miller and Corsellis, 1977; Baron et al., 1996; Cozzi et al., 2014; Povinelli et al., 2014). We are aware of the fact that the intraspecific variations of RB and EQ may alter our statistical results but this discussion can only be addressed when more data will be available in the future. The same holds true for the calculation of some biological parameters (e.g., activity type or dietary types). Here we developed generalized standardizations for these calculations (e.g., Table 1) because detailed data are missing for many species. Although represented by a complete matrix without missing values, our data set is restricted because some large orders, such as the Eulipotyphla, Hyracoidea, Lagomorpha, and Scandentia, are only occupied by two or three species (Figure 1). In this context, the expansion of database projects like PanTHERIA (Jones et al., 2009) would bring substantial new input into this genre of brain size studies.

Note that we analyzed separately various Eutheria species and did not take into account the phylogenetic origin and specialized adaptations, like echolocation or electroperception, in our statistical analysis. Here, comparisons of whole brain parameters (AB, RB, EQ) as a principle seems to be more appropriate than the comparison of selected ones such as brain regions, rhombomeres, circuits, or neuron types, which may show taxa-specific allometric relationships (Willemet, 2012). In this way, we avoided a separation of specific taxa with hypertrophic and rudimentary brain structures such as olfactory, auditory, visual or other parts of the brain. For instance, odontocetes have extended auditory nuclei, but no primary olfactory brain structures and small hippocampi, while primates have relatively large hippocampal structures and relatively smaller auditory nuclei (Oelschläger, 2008; Oelschläger et al., 2010; Shultz S. and Dunbar R. I. M., 2010).

Among parameters that could not be considered, the occurrence of menopause might be an interesting feature. However, only a few mammals live beyond their reproductive phase. Menopause had only been verified for two large dolphins (*Globicephala macrorhynchus, Orcinus orca*) and three primate species (*G. gorilla, Pan troglodytes, H. sapiens*; Johnstone and Cant, 2010). Even though menopause was not taken into account because it is so rare, it is striking to see that these species generally possess a large brain (Figures 2, 3). Hibernation was not included in the analysis for the same reason: among the 115 mammals examined, only two hibernate (*E. europaeus* and *Plecotus auritus*) and another two have periods of dormancy (*Ursus arctos* and *Thalarctos maritimus*; Puschmann, 1989).

### Brain size and the phylogenetic tree

The results of the cluster analysis show that, concerning the brain parameters AB, RB, and EQ, each group (cluster) includes Eutheria from phylogenetically distantly related taxa. Hence the whole brain parameters can be interpreted independently of the phylogenetic tree.

Interpreting the consequences of AB and RB differences in the respective orders, Stephan et al. (1988) described a secondary decrease or increase of the body mass along with a constant brain size and coined the terms dwarfism and giantism for closely related species. The phylogeny of the Equidae was discussed as an example of the increase of the body mass and a decreased RB, which largely aligns with the nutrition change from fruit and leaves to grass (Nowak and Paradiso, 1983; Wund and Myers, 2011). Cetacea also have disproportionally large body mass relative to brain mass, which might be explained by the modified gravitational forces in the aquatic habitat (Marino, 1998). Moreover, odontocetes as well as anthropoid primates have a greater variance in EQ, suggesting that evolutionary constraints resulting in a strict correlation between brain and body mass had become relaxed (Boddy et al., 2012; Montgomery et al., 2013). As a result, for some mammalian groups body mass appeared to be an inadequate reference value for the comparison of ungulates, marine mammals, and primates to other mammals (Radinsky, 1978; Manger et al., 2013). In our data, this constraint was especially evident in the example of the blue whale EQ, which was comparatively small (0.19) although the AB was high (~3.6 kg).

The primates were distributed into two separate groups (groups 4 and 5). Although their EQ values were similar to those of the old world monkeys from group 4, the fact that hominides (except man *H. sapiens*) were assigned to group 5 was probably due to their body mass. In this case, the RB was crucial for this classification. Because the interpretation of changes of RB and EQ is challenging when body masses change (Striedter, 2005), the use of the three measurements of brain mass (AB, RB, EQ) in parallel is more likely to discover general potential influences on brain mass. This fact is also shown by the discrete clustering of the Artiodactyla (groups 1 and 5) and Cetacea (groups 2 and 4) which belong to the same order Cetartiodactyla (Frey et al., 2015; Huggenberger and Klima, 2015).

### Brain size and critical biological parameters

**Figure 6:**
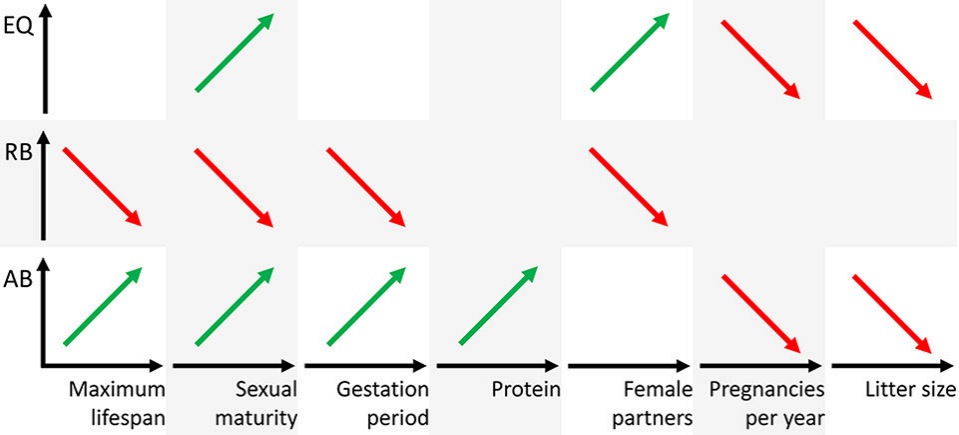
**Schematic representation of positive (green) or negative (red) correlations figuratively depicted as regression line of the three brain dimensions AB, RB, and EQ (ordinates) and the biological parameters (abscissas)**. Note that the parameters maximum lifespan, sexual maturity, and gestation period are time axes, the parameter protein is mass and the parameters female partners, pregnancies per year, and litter size are numbers.

The analyses of the various biological parameters for the five groups with the cluster analysis can be summarized as follows. The Eutheria with high AB in group 2 have long and rare pregnancies with small litter sizes, a late sexual maturity but long lifespans; they feed on protein-rich nutrition (Figures 4, 6). Animals with high RB such as Eutheria of group 3 have short and frequent gestation periods, an early sexual maturity, and a short lifespan. Additionally, males of group 3 have only few potential sexual partners (Figures 4, 6), whereas a high number of potential sexual partners is related to high EQs. Moreover, Eutheria with high EQ (group 4) show late sexual maturity and rare gestations with small litter sizes (Figures 4, 6).

Group 4, which was distinguished by a significantly high EQ, is comprised of three representatives from the group of new world monkeys: the white-headed capuchin *C. capucinus* that exhibits the ability of tool use and the spider monkeys *A. dariensis* and *A. geoffroyi* that use their tails to grab branches (Wilson and Reeder, 2005). Group 4 also includes three smaller representatives from the group of old world monkeys: the lar gibbon *Hylobates lar*, whose abilities to climb even exceeds those of a spider monkey (Nowak and Paradiso, 1983) and a larger representative from the group of old world monkeys, man *H. sapiens*, with the largest EQ. Interestingly, one predator, the kinkajou *P. flavus* from the family of Procyonidae, was also assigned to group 4. In this context it is also remarkable that, in contrast to the other Procyonidae, the ability of the kinkajou to climb using his tail to grab branches resulted in its accidental assignment to the group of lemurs when first described in 1774 (Nowak and Paradiso, 1983; Wund and Myers, 2011).

Former studies demonstrated that mammals with long gestation periods and long lifespans had large relative brain masses and EQs, respectively (Sacher and Staffeldt, 1974; Hofman, 1993). For instance, brain mass seemingly depended on the maternal energy available during gestation (Martin, 1981; Marino, 1998). In this study, only group 2, which was characterized by a significantly high AB, was separated in the discriminate analysis from the other groups on the basis of the gestation period. However, the average gestation period of group 2 must, apart from the brain size, also be considered in connection with other factors, such as BM, which in group 2 is above average (Figure 4A). On the one hand, another potentially important factor is that all representatives from group 2 are precocial (see Table S1). On the other hand, group 4, in which are also precocial mammals (except the kinkajou *P. flavus*), is similar to group 1 with regard to gestation periods, (Figure 4B). This pattern probably results from the fact that group 1 is comprised of a large number of Artiodactyla, which are precocial and have therefore the need of extended gestation periods (in comparison to groups 3 and 5) but low AB, RB, and EQ (Figure 4A). This may be why the gestation period was not a criterion to separate group 1 from the others in the course of the discriminant analysis. In our discriminant analysis, the frequency of pregnancies still reveals a tendency of an inverse correlation with the gestation periods (Figures 4B,F). The precocial Euteria from groups 2 and 4 (with relatively long gestation periods) were less often pregnant than representatives from group 3, which had short gestation periods and, apart from white-headed marmoset, was comprised of altricial animals. Up to now no correlations between the frequency of pregnancies or litter size (highest in groups 1 and 5; Figures 4F,G) and the brain mass of mammals had been observed. Gittleman (1986) for instance examined the brain size of carnivores with regard to litter size, but found no correlation. The author Gittleman (1986) pointed out that the different brain sizes between carnivores and insectivores might reflect the different complexities of their hunting strategies. While insectivores have to master smaller distances to find their prey and pick them with a relatively high success rate, carnivores must acquire complex hunting abilities and experience a comparably low success rate.

As mentioned above, differences in the biological parameters do not provide a robust explanation for the separation of groups 1 and 5 (cf. Figure 4). However, it was striking that group 5, whose representatives were predominantly omnivores or carnivores, also contained the Indian muntjac *M. muntjak* and the bay duiker *C. dorsalis* from the artiodactyls. In comparison to the artiodactyls from group 1 these animals show special features concerning their diet (see Table S1). The Indian muntjac feed on various kinds of plants, especially fruit, while the artiodactyls of group 1 were exclusively specialized on leaves and/or grass. Accordingly, the differences of brain parameters in groups 1 and 5 may reflect dietary differences that were not evident in our data. Only group 2 was separated from the other groups by the percentage of raw protein in the diet, but this parameter was not the sole factor for this separation.

It is generally plausible that various biological parameters may have co-evolved because they had been linked selection parameters. For example, selection favoring investment in a large number of offspring (r-selection) was shown to be correlated with small body size. Alternatively, K-selection (selection favoring investment in a small number of offspring to increase their fitness) promotes large body size, long lifespans, small litter sizes, and fewer descendants during lifetime (Pianka, 1970; van Dongen, 1998). Although these selection types (r- and K-selections) should be relevant for brain evolution (van Dongen, 1998) there is no distinct correlation to brain size and therefore interpretations are contradicting. Selection in favor of small body size may be correlated with larger EQs in some mammals and with the reduction of the EQ in other species (van Dongen, 1998).

## Conclusion

The brain is among the most cost-intensive organs with regard to metabolism. For this reason, a large brain has either to exhibit crucial survival benefits or it would have to surrender to selection pressure and ultimately reduce its size (Armstrong, 1982, 1983; Gibson, 1986; Aiello and Wheeler, 1995; Isler and van Schaik, 2009). Considering the statistical results from this study, it is important to note that our analyses are appropriate to correlate brain masses (AB, RB, EQ) with biological parameters such as physiology, development, ecology, and behavior. Implications of functional (cognitive) capabilities of the species were thus not possible. However, it is striking that the informative biological parameters (Figures 4, 6) can be used to define the potential trophic levels of eutherian species within their consumer-resource networks (Olff et al., 2009). This is where the lowest trophic levels of, for example, herbivores and insectivores from groups 1, 3, and 5 can be found; all of these animals have relatively short gestation periods along with a high frequency of pregnancies and large litter size, short lifespan, and early sexual maturity. Animals from groups 2 and 4, omnivores, carnivores, and piscivores that, despite long lifespans, give birth to few offspring because of late sexual maturity rank on higher trophic levels (Figure 4). Thus, a large AB (group 2) or a high EQ (group 4) is generally found in Eutheria of higher trophic levels. Animals of lower trophic levels only have a large RB if they are very small (small BM) like the Eutheria from group 3.

## Author contributions

CS and LZ contributed equally as first authors. Conception of study: CS, LZ, KM, SH. Design of study: CS, LZ, MH, WW, SH. Data collection: CS, LZ, MH. Data Analysis: CS, LZ, KM. Interpretation of data: CS, LZ, MH, KM, WW, SH. Discussion of literature: CS, LZ, MH, KM, WW, SH.

### Conflict of interest statement

The authors declare that the research was conducted in the absence of any commercial or financial relationships that could be construed as a potential conflict of interest.
