# Supplementary material for: Multivariate Meta-Analysis of Brain-Mass Correlations in Eutherian Mammals
Source: Front Neuroanat. 2016 Sep 30;10:91. doi: 10.3389/fnana.2016.00091 (PMC5043137; doi:10.3389/fnana.2016.00091)
Supplement: Supplementary file 1 [file DataSheet1.pdf]

Table S1: Species and raw data of biological parameters used in the analysis (data references see Material and Methods). AT, activity period in hours during daytime (circadian activity); BM, body mass (g); EO, period of postnatal eye opening in days (eye opening); FP, mean number of female sexual partners during a ten years period (female partners); FT, food type (Table 5); GP, gestation period in days; GS, group size; LS, litter size; MA, maximum lifespan in years; MP, mean number of male sexual partners during a ten years period (male partners); NP, nursing period in days; PY, frequency of pregnancies (pregnancies per year); SM, sexual maturity in days.

Species	AT	EO	FP	FT	GS	GP	LS	MA	MP	NP	PY	SM
Ammotragus lervia	0	0	19.0	6	20	158	1.2	4380	1	120	1.00	730
Antilope cervicapra	12	0	27.0	7	28	120	1.0	6570	1	60	1.00	730
Axis axis	6	0	5.0	8	10	218	2.0	7300	5	180	1.00	383
Bos grunniens	12	0	7.0	8	8	255	1.0	9125	1	180	1.00	685
Bos taurus taurus	12	0	7.0	6	8	280	1.0	7300	1	300	1.00	545
Capra hircus	12	0	19.0	7	20	151	2.0	5475	1	300	0.67	288
Cephalophus dorsalis	0	0	1.0	10	2	120	1.0	2190	1	120	2.00	457
Cervus canadensis	3	0	19.0	8	20	255	1.0	5475	1	180	1.00	867
Cervus elephus	6	0	19.0	8	20	235	1.0	5475	1	180	1.00	785

Cervus unicolor	0	0	4.0	8	5	246	1.1	8213	1	365	1.00	913
Choeropsis liberiensis	0	0	1.0	7	1	205	1.0	13870	1	280	0.13	1593
Dama dama	12	0	29.0	8	30	240	1.0	6753	1	365	1.00	959
Hippopotamus amphibius	6	0	10.0	7	20	240	1.0	12775	10	365	1.00	3240
Lama glama	12	0	19.0	7	20	330	1.0	9125	1	120	1.00	730
Muntiacus muntjac	6	0	10.0	4	1	183	1.1	6205	10	120	1.00	360
Ovis aries	12	0	30.0	7	300	150	2.4	5110	1	180	1.00	730
Rangifer tarandus	12	0	10.0	8	55	225	1.0	4928	1	45	1.00	730
Sus scrofa	6	0	19.0	4	20	120	4.0	2738	1	105	1.00	500
Sus scrofa domestica	12	0	19.0	4	20	115	8.0	4380	1	105	2.00	500
Taurotragus oryx	3	0	10.0	8	20	255	1.0	8213	10	150	1.00	1278
Vicugna vicugna	12	0	7.0	7	8	300	1.1	8760	1	300	1.00	1826
Ailuropoda melanoleuca	6	179	10.0	6	1	143	1.5	10950	10	165	1.00	2373
Ailurus fulgens	0	26	1.0	10	2	120	1.5	4928	1	158	1.00	540
Callorhinus ursinus	0	0	17.5	2	1	254	1.0	5753	1	105	1.00	1552
Canis latrans	3	12	1.0	3	2	62	7.5	7939	1	63	1.00	645

Canis lupus	6	14	1.0	3	7	63	8.0	5475	1	63	1.00	645
Canis lupus familiaris	12	14	1.0	3	2	63	4.0	3285	1	42	2.00	270
Hyaena hyaena	0	7	10.0	4	1	90	4.5	8760	10		2.00	365
Leptonychotes weddellii	12	0	10.0	2	1	310	1.0	7300	1	46	1.00	2191
Lynx canadensis	0	11	10.0	1	1	60	2.5	4015	10	150	1.00	810
Mungos mungo	12	10	7.5	5	15	60	3.2	3011	8	730	0.25	730
Otocyon megalotis	0	5	1.0	3	2	65	4.0	1825	1	105	1.00	365
Pagophilus groenlandicus	12	0	10.0	2	1	240	1.0	10950	10	14	1.00	2008
Panthera leo	3	3	12.0	1	13	108	3.0	6205	1	195	0.55	1278
Panthera pardus	6	9	10.0	1	1	92	2.5	8395	10	90	0.50	1095
Panthera tigris	6	9	10.0	1	1	104	3.0	9490	10	135	0.25	1461
Potos flavus	0	17	1.0	4	3	77	1.2	4745	1	56	1.00	685
Procyon lotor	0	21	10.0	4	20	63	4.0	1095	10	120	1.00	457
Suricata suricatta	12	13	1.0	5	30	74	3.0	4563	1	730	1.00	365
Taxidea taxus	0	35	10.0	3	1	60	2.5	5110	10	42	1.00	456
Thalarctos maritimus	12	34	10.0	3	1	240	1.8	10038	10	730	1.00	2008

Urocyon littoralis	6	14	1.0	3	2	63	3.7	2920	1	56	1.00	300
Ursus arctos	1	32	10.0	4	1	219	2.3	9125	10	730	0.33	1734
Vulpes vulpes	6	14	1.0	3	1	52	4.0	4380	1	42	1.00	300
Zalophus californianus	12	0	16.0	2	17	343	1.0	7300	1	270	1.00	1917
Balaenoptera musculus	6	0	10.0	2	1	330	1.0	18250	10	195	0.50	2190
Eschrichtius robustus	6	0	10.0	2	1	398	1.0	18250	10	225	0.50	2920
Megaptera novaeangliae	6	0	3.5	2	7	345	1.0	12775	4	150	0.50	1643
Orcinus orca	12	0	13.3	2	27	495	1.0	14600	13	540	0.22	3559
Phocoena phocoena	6	0	3.8	2	8	270	1.0	3285	4	225	0.66	1096
Physeter macrocephalus	6	0	16.5	2	18	450	1.0	18250	1	730	0.20	2920
Pseudorca crassidens	6	0	9.0	2	18	405	1.0	21900	9	630	0.14	3376
Stenella attenuata	12	0	50.0	2	100	341	1.0	16425	50	600	0.40	4289
Tursiops truncatus	12	0	30.0	2	60	360	1.0	10950	30	540	0.20	3833
Artibeus jamaicensis	0	4	7.0	9	8	75	1.0	3650	1	60	2.00	408
Desmodus rotundus	0	4	30.0	1	60	213	1.0	4836	30	270	2.00	365
Nyctalus noctula	1	4	10.0	5	100	75	2.0	3650	1	60	1.00	228

Plecotus auritus	0	4	6.0	5	8	75	1.0	3650	1	42	1.00	365
Dendrohyrax arboreus	0	0	1.0	6	30	225	1.5	4471	1	90	1.00	510
Procavia capensis	12	0	40.0	10	80	225	3.0	4015	40	120	1.00	510
Lepus europaeus	1	0	10.0	10	1	41	2.5	1825	10	28	4.00	195
Oryctolagus cuniculus	3		15.0	10	30	31	5.0	3285	15	28	6.00	90
Erinaceus europaeus	1	16	10.0	5	1	34	4.6	1095	10	42	1.00	270
Elefantulus myurus	12	13	1.0	5	2	46	1.5	548	1	18	3.50	45
Elephantulus intufi	12	13	1.0	5	2	51	1.5	548	1	25	3.50	50
Equus asinus	12	0	11.5	7	13	330	1.0	8030	1	210	1.00	1004
Equus caballus	12	0	11.5	7	13	330	1.0	10950	1	210	0.50	594
Equus quagga granti	12	0	11.5	7	13	371	1.0	8395	1	210	0.50	1004
Rhinoceros bicornis	12	0	10.0	6	1	452	1.0	16425	10	540	1.00	2008
Tapirus indicus	0	0	10.0	6	1	390	1.0	10950	10	365	0.50	1278
Alouatta palliata	12	0	14.0	10	15	139	1.0	7300	1	300	0.53	1552
Ateles dariensis	12	0	2.5	4	5	140	1.0	12045	3	420	0.33	1643
Ateles geoffroyi	12	0	2.5	4	5	140	1.0	7300	3	450	0.33	1643

Callithrix geoffroyi	12	0	1.0	4	9	145	2.0	5840	1	60	0.50	450
Cebus capucinus	12	0	9.5	4	19	180	1.0	7300	10	90	0.50	1860
Cercopithecus pygerythrus	12	0	24.0	4	25	195	1.0	10585	1	180	1.00	1278
Gorilla gorilla	12	0	15.0	6	16	265	1.0	19710	1	1278	0.25	3468
Homo sapiens	12	0	1.0	4	2	270	1.0	27375	1	365	0.66	4380
Hylobates lar	12	0	1.0	9	2	210	1.0	9125	1	540	0.44	3103
Lemur catta	12	0	17.0	10	18	125	1.5	6570	1	150	1.00	756
Macaca mulatta	12	0	47.0	4	94	175	1.0	12045	47	365	0.50	1643
Nycticebus coucang	0	0	10.0	4	1	190	1.2	4745	10	270	1.75	525
Otolemur crassicaudatus	0	0	1.0	4	4	135	1.0	6570	1	150	1.00	730
Pan troglodytes	12	0	27.5	10	55	230	1.0	21900	28	1460	0.33	2555
Papio hamadryas	12	0	7.3	4	100	180	1.0	13505	1	239	1.00	2464
Pongo pigmaeus	12	0	10.0	9	1	270	1.0	21170	1	1278	0.29	3650
Presbytis obscurus	12	0	17.0	6	18	168	1.0	9125	1	540	0.50	1917
Elephas indicus	6	0	19.0	8	20	630	1.0	25550	1	270	0.29	3741
Loxodonta africana	6	0	7.0	8	8	655	1.0	21900	1	270	0.20	3650

Castor canadensis	0	0	1.0	10	6	128	2.5	6570	1	90	1.00	1278
Castor fiber	0	0	1.0	10	12	128	3.0	4928	1	90	1.00	1186
Cavia porcellus	6	0	3.8	10	8	64	2.6	2920	4	21	5.00	75
Chinchilla laniger	0	0	1.0	10	2	110	2.0	5475	1	49	2.50	240
Cricetulus griseus	0	12	10.0	4	1	21	4.6	730	10	20	3.50	50
Dasyprocta aguti	12	0	1.0	9	2	104	1.3	6205	1	140	2.00	180
Gerbillus pyramidum	1	12	10.0	4	1	21	4.0	730	10	30	7.00	120
Glaucomys volans	3	25	10.0	4	1	40	3.1	4380	10	63	2.00	270
Hydrochoerus hydrochaeris	1	0	12.0	10	13	123	3.0	3285	1	105	1.00	450
Hystrix cristata	0	7	1.0	10	7	112	1.2	4928	1	60	2.00	365
Mesocricetus auratus	0	12	10.0	4	1	16	6.3	913	10	20	2.00	53
Mus musculus	0	13	7.5	4	15	19	5.0	730	8	21	7.50	42
Myocastor coypus	6	0	1.0	10	2	132	5.5	2190	1	42	2.50	100
Peromyscus gossypinus	0	13	10.0	4	1	23	3.7	730	10	25	3.00	40
Peromyscus leucopus	0	16	10.0	4	1	23	3.5	730	10	25	2.00	40
Peromyscus maniculatus	0	15	3.5	4	7	24	5.0	730	4	30	7.00	49

Rattus natalensis	0	16	22.5	4	45	23	7.3	1460	23	22	7.00	88
Rattus norvegicus	1	14	27.5	4	55	21	8.0	365	28	22	6.00	113
Sciurus vulgaris	12	31	10.0	4	1	38	3.0	1460	10	63	2.00	365
Tupaia glis	12	7	1.0	4	2	46	1.5	913	1	35	1.00	131
Tupaia minor	12	17	1.0	4	2	55	1.5	4198	1	38	1.00	60
Urogale everetti	12	17	1.0	4	2	50	2.0	4198	1	38	1.00	90
Bradypus griseus	12	0	10.0	6	1	173	1.0	3285	10	180	1.00	1004
Choloepus hoffmanni	12	0	10.0	10	1	173	1.0	11680	10	30	1.00	1004
Dasypus novemcinctus	0	0	10.0	5	1	120	4.0	5110	10	135	1.00	365
Tamanduas tetradactyla	0	0	10.0	5	1	180	1.0	3468	10	130	1.00	730

Tables S2 to S5: Summary of canonical discriminant functions of the normalized three brain parameters absolute brain mass (AB), relative brain mass (RB), and encephalization quotient (EQ)

Table S2: Eigenvalues

Function	Eigenvalue	% of variance	Canonical correlation
1	6,803[a]	55,4	0,934
2	4,351[a]	35,5	0,902
3	1,116[a]	9,1	0,726

Table S3: Wilks‘ Lamda

Test of functions	Wilks-Lambda	Chi-square	Degrees of freedom	Significance
1 bis 3	0,011	492,952	12	0,000
2 bis 3	0,088	266,958	6	0,000
3	0,473	82,459	2	0,000

Table S4: Standardized canonic discriminant function coefficients

	Function 1	Function 2	Function 3
AB	0,821	0,587	-0,108
RB	-0,484	0,862	-0,211
EQ	-0,090	0,088	1,003

Table S5: Classification results after Ward (*92,2% of grouped individuals were correctly reclassified)

	Ward Method	Sum*
Original	Cluster 1	52 (100%)
	Cluster 2	32 (100%)
	Cluster 3	14 (100%)
	Cluster 4	8 (100%)
	Cluster 5	9 (100%)
Cross validation	Cluster 1	52 (100%)
	Cluster 2	32 (100%)
	Cluster 3	14 (100%)
	Cluster 4	8 (100%)
	Cluster 5	9 (100%)
